# Correlation Between Serum Alpha-Fetoprotein and Tumour Size in Patients With Hepatocellular Carcinoma Treated With Direct-Acting Antivirals

**DOI:** 10.7759/cureus.24506

**Published:** 2022-04-26

**Authors:** Daniel Rusie, Adriana Mercan Stanciu, Letitia Toma, Elena Laura Iliescu

**Affiliations:** 1 Department of General Surgery, Clinical Emergency Hospital, Bucharest, ROU; 2 Department of Internal Medicine II, Fundeni Clinical Institute, Bucharest, ROU; 3 Department of Internal Medicine, Carol Davila University of Medicine and Pharmacy, Bucharest, ROU

**Keywords:** hepatitis c virus (hcv), tumour burden score, direct-acting antivirals, hepatocellular carcinoma, serum alpha-fetoprotein

## Abstract

Objectives: Since its first description, alpha-fetoprotein has become the most widely used marker for diagnosing and monitoring patients with hepatocellular carcinoma (HCC). This study aims to assess the correlation between serum levels of alpha-fetoprotein and tumour dimensions in patients diagnosed with HCC, that were previously treated with direct-acting antivirals for hepatitis C viral infection.

Materials and methods: We conducted a retrospective cohort study on 47 patients with a personal history of hepatitis C virus infection, who were diagnosed with different forms of HCC more than one year after achieving sustained virologic response after 12 weeks post-treatment. Patients were monitored by liver function tests, tumoral markers, blood cell count and coagulation profile and underwent imagistic explorations such as abdominal ultrasonography and, in selected cases, computerised tomography/magnetic resonance imaging. Tumour burden was assessed by both tumour burden score and seven-eleven criteria.

Results: The study mostly included cirrhotic patients, multinodular HCC being the predominant pattern. All patients had alpha-fetoprotein levels over 100 ng/ml, with values largely varying, in accordance with the tumour dimensions. Most patients had medium-range Tumour Burden Score, a variable that also correlated with nodule size.

Conclusions: The study found a significant correlation between serum alpha-fetoprotein and tumour size in patients with HCC. Alpha-fetoprotein also correlated well with Tumour Burden Score and remains a very important diagnostic and prognostic tool for patients with HCC.

## Introduction

Hepatocellular carcinoma (HCC) is the sixth most frequently encountered malignancy in the world and the fourth major cause of cancer-related mortality worldwide [1]. According to the most recent estimations of The World Health Organization, more than 1 million individuals are expected to die due to hepatic cancer in 2030 [2]. Viral hepatitis has always represented an important risk factor in the development of HCC, with hepatitis C virus (HCV) being associated with a significant increase (up to 20-fold) in HCC occurrence [2,3]. Although in this new era of direct-acting antivirals (DAAs), there has been significant progress towards eradicating HCV, antiviral agents do not completely obliterate the risk for HCC, thus HCC screening should be thoroughly continued in all patients with severe fibrosis (F3) and cirrhosis (F4) [2,4].

Alpha-fetoprotein (AFP) is an oncofoetal glycoprotein produced by the yolk sac, foetal liver and gastrointestinal tract during intrauterine and neonatal life [5]. Since its first description in 1953, AFP turned into the most used tumoral marker for HCC diagnosis and surveillance, including treatment response assessment [6]. In patients considered at risk for developing this type of cancer, AFP represents a powerful screening tool, combined with abdominal ultrasonography [6,7]. However, there may be variation in AFP sensitivity and specificity, in relation to patient characteristics and cut-off values [5,8]. While a decrease in the cut-off value for AFP can improve the sensitivity, this may lead to false-positive results. Similarly, increasing the cut-off may result in specificity enhancement, but with a reduction in sensitivity [5,8,9].

Although the AFP-HCC association is clear, in almost one-third of the cases, HCC presents with normal AFP levels [5]. These situations have been associated with better outcomes and a smaller probability of recurrence, in comparison to the patients with high AFP levels [5,10]. Elevated AFP can also be encountered in other types of malignancies, as well as in chronic liver disease, in the absence of HCC (due to liver regeneration, inflammation and fibrosis) [5,11]. Moreover, literature data suggest that AFP could be a significant prognosis marker in patients with HCV chronic infection, regardless of HCC presence [12].

This study aims to assess the correlation between serum levels of alpha-fetoprotein and tumour dimensions in HCC patients, that were previously treated with DAAs for hepatitis C viral infection, obtaining sustained virologic response (SVR).

## Materials and methods

We conducted a retrospective observational study, on 47 patients with HCC, that had a prior history of HCV infection, for which they received treatment with DAAs (either ritonavir-boosted paritaprevir/ombitasvir and dasabuvir or ledipasvir/sofosbuvir). The subjects were admitted to our clinic between January 2018 and December 2021. Antiviral therapy was given to each patient, in accordance with the National Healthcare Program available at that moment. The local ethical committee approved the study. Informed written consent was taken from all the participants and all their records were confidential.

We included in the study patients with personal history of HCV chronic infection, who achieved SVR through DAAs. SVR was defined as undetectable HCV ribonucleic acid (HCV-RNA) at 12 weeks after ending direct antiviral therapy. All subjects were diagnosed with de novo HCC-documented by either computerised tomography (CT) or magnetic resonance imaging (MRI). No subject underwent liver biopsy for HCC diagnosis, due to its invasive character and susceptibility to associated complications. It is worth mentioning that for all patients included in the study, HCC was diagnosed more than one year after SVR achievement. We would like to emphasise on the fact that all patients with prior HCV infection should be routinely monitored every six months, in the presence of advanced fibrosis (F3) or cirrhosis, even after SVR is obtained [13]. No therapeutic measure targeting HCC was performed for any of the patients by the moment they were included in the study. We excluded the patients with concomitant or previous malignant processes, autoimmune liver disorders, heart failure, hepatitis B virus or human immunodeficiency virus (HIV) co-infection.

Demographic profiles were recorded. Patients’ evaluations also included liver function tests, renal function monitoring, blood cell count, and coagulation profile. Serum AFP level was determined using standard ELISA kits, by strictly following the manufacturer’s instructions. The AFP reference range provided by our laboratory was between 1.3 and 8 ng/ml. Based on the AFP blood levels, patients were divided into three subgroups: 100-300 ng/ml, 300-500 ng/ml and over 500 ng/ml. Liver fibrosis was evaluated by Fibroscan® (Echosens™, Paris, France). There were no F0, F1 or F2 among the study group. When stratifying patients, we considered a cut-off value of 9.6 kPa to be suggestive for F3, while values higher than 12.5 kPa defined F4 fibrosis.

Comorbidities such as diabetes mellitus, metabolic syndrome or hypothyroidism were also noted and monitored, when present. The albumin-bilirubin (ALBI) score was calculated for each subject, in order to assess liver function in patients with HCC, using the well-known formula: (log10 bilirubin (µmol/l) × 0.66) + (albumin (g/l) × −0.0852). The cut points of the ALBI grade were as follows: ≤ −2.60 (ALBI grade 1), from −2.60 to −1.39 (ALBI grade 2) and > −1.39 (ALBI grade 3) [14].

All patients underwent abdominal ultrasonography, as well as either CT or MRI for further characterisation of HCC, describing important tumour parameters such as the number of nodules, maximum nodule dimensions and position. Tumour burden was evaluated using tumour burden score (TBS), a parameter that incorporates both number and size into a single variable, according to the following formula: √(maximum tumour diameter^2^ + number of nodules^2^). Patients were divided into three groups based on TBS: high (over 13.74), medium (3.36-13.74) and low TBS (less than 3.36). We also took into consideration the newly proposed seven-eleven criteria (SEC), which calculates tumour burden in a different manner than TBS, as the sum of tumour number and maximum tumour diameter [15]. According to the SEC, high tumour burden is defined as >11, intermediate tumour burden as >7 but no more than 11 and low tumour burden is less than 7. Data were analysed using the SPSS 18.0 statistical software (SPSS Inc., Chicago, IL, USA). Numerical values were expressed as mean +/- standard deviation.

## Results

We collected and analysed data from 47 patients diagnosed with HCC, with a personal history of DAAs treatment for HCV chronic infection. Forty-three of them had F4 fibrosis (including 26 patients with decompensated cirrhosis) and four patients presented with F3 fibrosis. Almost 60% of the patients were females. Cirrhotic patients were predominant (with over 55% already having decompensated cirrhosis at the moment of HCC diagnosis). Further baseline characteristics are presented in Table 1.

**Table 1:** Baseline characteristics of the study group ALBI: albumin-bilirubin; SEC: seven-eleven criteria; AFP: alpha-fetoprotein; HCC: hepatocellular carcinoma; TBS: tumour burden score

Total number of patients (47)	F4 (43 patients—91.49%)				F3 (4 patients—8.51%)	
	Decompensated cirrhosis (26 patients—55.32%)		Compensated cirrhosis (17 patients—36.17%)			
	Solitary HCC nodule N=6 patients (23%)	Multinodular HCC N=20 patients (77%)	Solitary HCC nodule N=2 patients (11.76%)	Multinodular HCC N=15 patients (88.23%)	Solitary HCC nodule N=3 patients (75%)	Multinodular HCC N=1 patient (25%)
Mean age	48.23+/- 15.02	53.83 +/- 17.45	51.21 +/- 21.83	51.87 +/- 16.75	48.33 +/- 18.13	53.78 +/- 21.05
Gender—female (27 patients—57.44%)	3 patients (50%)	12 patients (60%)	1 patient (50%)	9 patients (60%)	1 patient (33.33%)	1 patient (100%)
AFP=100-300 ng/ml (18 patients—38.29%)	1 patient (3.84%)		13 patients (76.47%)		4 patients (100%)	
AFP=300-500 ng/ml (17 patients—36.17 %)	15 patients (57.69%)		2 patients (11.76%)		0	
AFP > 500 ng/ml (12 patients—25.53%)	10 patients (38.46%)		2 patients (11.76%)		0	
Mean AFP (ng/ml)	660		278		250	
Mean HCC size (cm)	5.8		3.5		3	
Low TBS (13 patients—27.65%)	0		9 patients (52.94%)		4 patients (100%)	
Medium TBS (34 patients—72.34%)	26 patients (100%)		8 patients (47.06%)		0	
High TBS	0		0		0	
Low SEC (31 patients—65.96%)	12 patients (46.15%)		15 patients (88.24%)		4 patients (100%)	
Medium SEC (16 patients—34.04%)	14 patients (53.84%)		2 patients (11.76%)		0	
High SEC	0		0		0	
ALBI grade 1 (4 patients—8.51%)	0		0		4 patients (100%)	
ALBI grade 2 (30 patients—63.82%)	13 patients (50%)		17 patients (100%)		0	
ALBI grade 3 (13 patients—27.65%)	13 patients (50%)		0		0	

**Figure 1:**
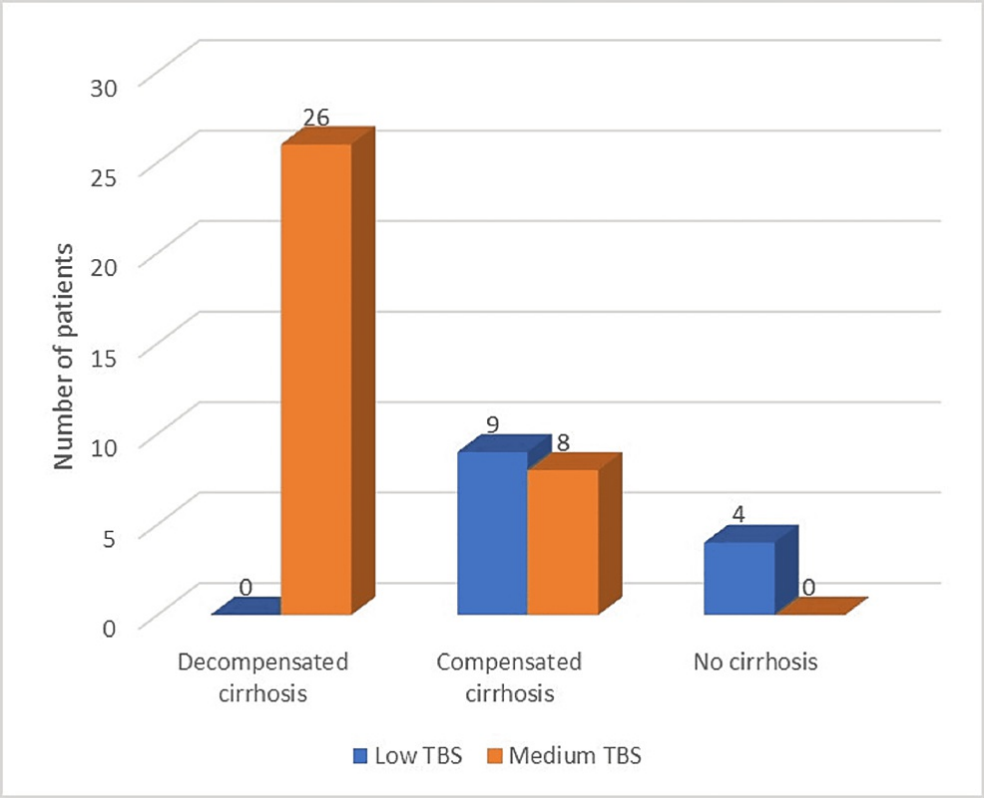
Distribution of study group by liver function and TBS TBS: tumour burden score

**Figure 2:**
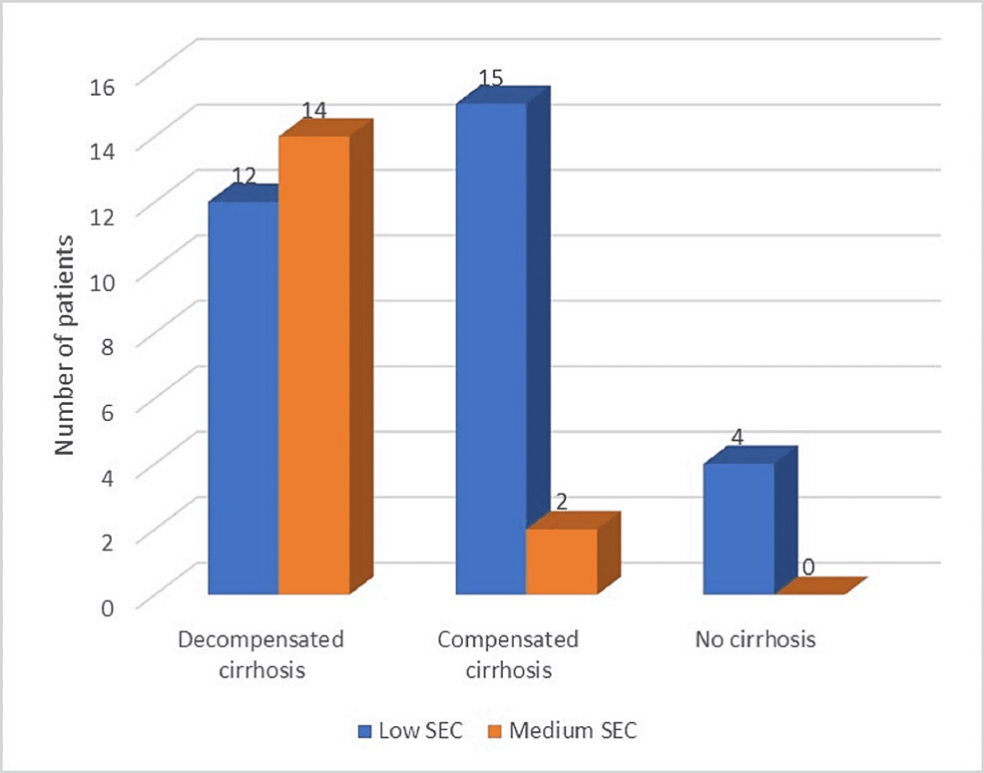
Distribution of study group by liver function and SEC SEC: seven-eleven criteria

Regarding the HCC pattern, within the total group of patients, more than 75% had multinodular HCC, while the rest had solitary nodules. The multinodular pattern was predominant within the cirrhotic patients, while non-cirrhotic patients mainly developed solitary hepatocarcinoma nodules. No patient with HCC included in our study had high TBS or high SEC at the moment of diagnosis (Figures 1-2). The most significant TBS grading was 9.06 (a value that represented medium-range TBS). Moreover, medium TBS was predominant within the total group of subjects, as well as within the cirrhotic group (34 out of the 43 cirrhotic patients, representing almost 80%). We have also observed that all patients with medium TBS had established cirrhosis. Also, all patients with decompensated cirrhosis and HCC had medium TBS. Only four patients were non-cirrhotic (but had F3 fibrosis): within this category, single-nodule HCC was predominant (75%) and all patients had low range TBS. Noticeable, all F3 patients also had other important risk factors for HCC development, besides HCV history (diabetes, hypothyroidism, metabolic syndrome).

**Figure 3:**
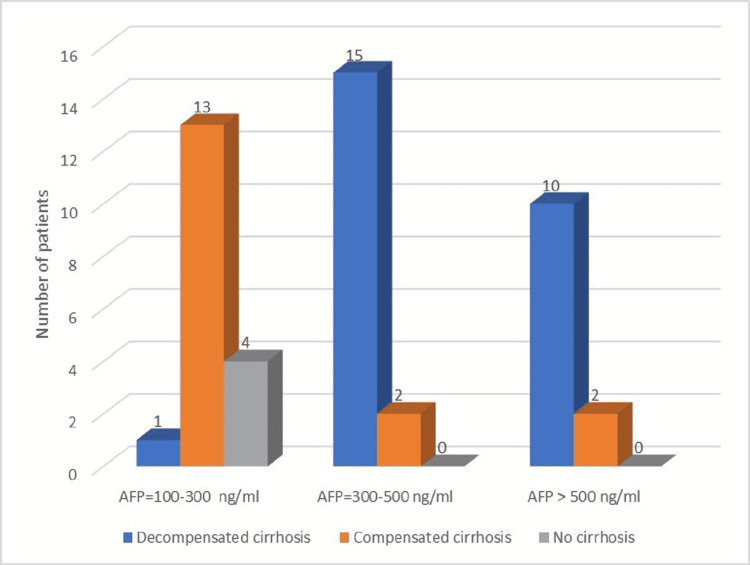
Distribution of study group by liver function and AFP AFP: alpha-fetoprotein

**Figure 4:**
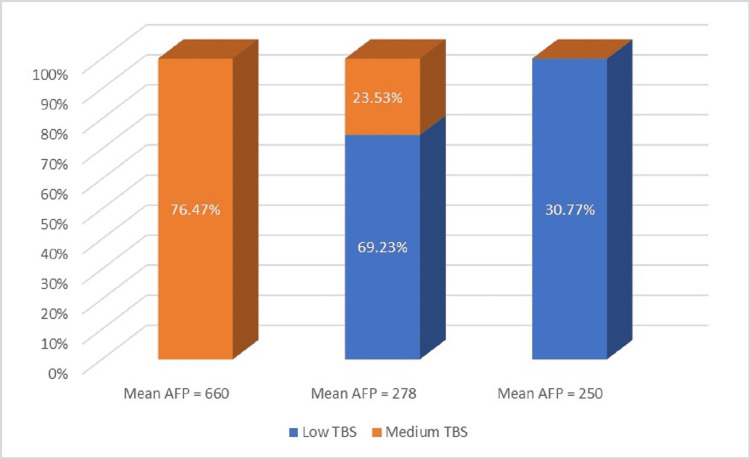
Mean AFP and TBS AFP: alpha-fetoprotein; TBS: tumour burden score

On the other hand, calculation of tumour burden by SEC resulted in a predominance of subjects with low SEC (almost 66%), including the four non-cirrhotic patients, while medium SEC was observed in 34% of the patients (Table 1). Most patients had AFP levels between 100 and 500 ng/ml, while approximately 25.5% presented AFP values higher than 500 ng/ml (Figure 3). All patients with AFP over 500 ng/ml were cirrhotic, with a predominance of decompensated cirrhosis and multinodular HCC pattern. Mean AFP among patients with decompensated cirrhosis was 660 ng/ml (Figure 4) and the mean HCC size for this category was 5.8 cm, with maximum tumour diameter ranging between 3.3 and 8.2 cm. Multinodular HCC was predominant within this subgroup and the maximum number of nodules was three. Objectivation of liver function through the ALBI score resulted in 50% patients with ALBI grade 2 and 50% patients with ALBI grade 3.

Patients with HCC and compensated cirrhosis had mostly AFP levels between 100 and 300 ng/ml, with a mean value of 278 ng/ml. Almost 90% of the patients included in this subgroup had multiple HCC nodules (the maximum number of nodules was three). The mean tumoral dimension was 3.5 cm, with the maximum tumour diameter ranging between 1.8 and 6.8 cm. The distribution of low TBS vs medium TBS was similar in patients with compensated cirrhosis (53% vs 47% representing nine vs eight patients), while SEC calculation resulted in a very different characterisation of the tumour burden in these patients, with almost 90% having low SEC. All patients with compensated cirrhosis and HCC had an ALBI score that corresponded to grade 2.

Patients with HCC and F3 fibrosis, which represented a small subgroup in this study, had mainly solitary nodules, with a mean HCC size of 3 cm and a mean AFP of 250 ng/ml. Within this category of subjects, TBS correlated well with SEC, resulting only in patients with low tumour burden. All F3 patients were ALBI grade 1. Detailed information regarding AFP levels, HCC size, ALBI score and tumour burden is presented in Table 1. The correlation of serum AFP levels with tumour sizes is detailed in Table 2.

**Table 2:** Correlation of serum AFP levels with tumour dimensions AFP: alpha-fetoprotein; HCC: hepatocellular carcinoma

	AFP=100-300 ng/ml	AFP=300-500 ng/ml	AFP > 500 ng/ml	P-value
Number of patients (total = 47 patients)	(18 patients—38.29%)	(17 patients—36.17%)	(12 patients—25.53%)	
Decompensated cirrhosis (26 patients—55.32%), mean HCC size = 5.8 cm	1 patient (3.84%)	15 patients (57.69%)	10 patients (38.46%)	0.002
Compensated cirrhosis (17 patients—36.17%), mean HCC size = 3.5 cm	13 patients (76.47%)	2 patients (11.76%)	2 patients (11.76%)	0.040
F3 (4 patients—8.51%), mean HCC size = 3 cm	4 patients (100%)	0	0	0.046

All patients were evaluated by CT scan and/ or MRI. Twelve patients underwent both CT and MRI evaluation and, in three of these cases, the lesions described in MRI turned out to be larger than CT estimated. In these scenarios, both TBS and SEC were calculated using the higher variables (as described by MRI). Typical MRI findings were hypo-intensity on T1-weighted images and T2 hyperintensity. Also, three of the cirrhotic patients with multinodular HCC presented portal vein thrombosis (PVT)-a fact that emphasises on tumour aggressiveness, suggesting a poor prognosis, while also being consistent with previous findings of portal vein thrombosis associated with larger tumours and higher AFP levels.

## Discussion

The aim of this paper was to observe and describe the correlation between serum AFP level and tumour size in patients with HCC, that previously received DAAs therapy for HCV infection. The development of DAAs opened a new era in the management of HCV-induced liver disease. However, the relationship between the use of DAAs and HCC has been the subject of numerous controversies, with several authors suggesting that interferon-free therapy is associated with more rapid tumour growth, resulting in a higher incidence of HCC [16-18]. On the other side, there has been emerging evidence that found no link between HCC occurrence and DAA therapy [18]. Regarding our cohort of 47 people, HCC was only found in the presence of cirrhosis and advanced fibrosis (F3), conditions that represent independent risk factors for HCC development. Moreover, it is noticeable that some of the subjects included in the study also presented with diabetes mellitus, metabolic syndrome or hypothyroidism, pathologies that are also known to be involved in HCC development [19]. It is important to emphasise that HCC screening (using imagistic methods and tumour markers every six months) should not be stopped in patients with severe fibrosis (F3) and cirrhosis (F4), after the achievement of SVR, as interferon-free treatments cure the viral infection, but not the liver disease itself. The study resulted in a predominance of multinodular HCC, a predictable pattern as most of the patients (43) were cirrhotic (including 26 individuals with decompensated cirrhosis) and thus had advanced fibrosis. The four non-cirrhotic patients included in the study also had an important degree of fibrosis (F3), but they mainly developed solitary hepatocarcinoma nodules.

Serum AFP is frequently increased in the presence of HCC and within our study group, we did not encounter AFP levels below 100 ng/ml, suggesting once again that AFP represents a powerful screening tool for this type of cancer. Although normal-AFP HCC is considered to have a better outcome [5], increased AFP levels at diagnosis (exceeding laboratory limits) allow the clinician to assess prognosis, as well as treatment responsiveness more easily. Thus, a complete response to the treatment should be expected if previous levels of AFP were lower than normal values on follow-ups, while a novel elevation of AFP after treatment may suggest tumour recurrence or metastatic disease [20]. AFP assessment is important as detection of small tumours or metastases can at times be difficult on routine imagistic evaluation. Moreover, even the use of advanced imaging methods such as CT may result in an incomplete characterisation of tumoral lesions, especially in infiltrative patterns of HCC. Within our study group, we found three cases where the lesions identified by the MRI were larger and more extensive than the CT scan had anticipated. While definitory HCC features can be observed on both multiphasic CT and MRI, the use of MR, although not always cost-effective, has the advantage of offering additional imaging sequences and is capable of distinguishing subtle lesions, ensuring a more accurate characterisation of HCC patterns and dimensions.

Many authors have evaluated the relation between AFP levels and HCC size. Saffroy et al. [21] discovered that larger tumours presented with higher AFP levels, while a wide proportion of small HCCs (80%) had normal AFP. The sensitivity of this marker was higher when nodules exceeded 3 cm in diameter (52% compared to 25% in tumours below 3 cm) [21].

Within our study, we observed that, in patients with decompensated cirrhosis, where the mean HCC size was 5.8 cm (maximum tumour diameter ranging between 3.3 and 8.2 cm), AFP levels were mostly over 300 ng/ml, with many values exceeding 500 ng/ml and with a mean AFP of 660 ng/ml. On the other hand, the rest of the DAAs-treated patients (both compensated cirrhosis and F3 hepatitis) presented with significantly smaller tumours (mean HCC size being 3.5 and 3 cm, respectively) and had lower AFP levels, mostly between 100 and 300 ng/ml-in accordance to their dimensions. The data we obtained are in agreement with other authors’ findings, showing that, when elevated, AFP values are in correlation with HCC size.

As we have already mentioned, elevated AFP can also be explained by the existence of advanced chronic disease and it is true that the subjects who presented the higher AFP levels in our study also had decompensated cirrhosis. But cirrhotic liver was also a feature among the second subgroup of patients, that presented with smaller tumours and lower AFP. This fact strongly suggests that, when HCC is present, AFP levels correlate with tumour size, regardless of the degree of fibrosis. For tumour burden estimation, we used both TBS and SEC, resulting in different distributions: most patients had medium TBS, but low SEC. The most significant TBS grading was 9.06, a value that represented medium-range TBS, with no patient having either high TBS or high SEC at the moment of diagnosis. This may suggest that after DAAs treatment, HCC is less aggressive, even in patients with advanced cirrhosis. However, this affirmation should be further investigated, in larger cohorts.

We observed a correlation between TBS and AFP levels, as an important proportion of medium TBS patients had AFP levels over 300 ng/ml, while low TBS was mainly associated with AFP under 300 ng/ml. Both TBS and AFP are strong prognosis factors in patients diagnosed with HCC and the fact that these variables are in relation to one another allows the clinician to create a stronger prediction regarding the possible outcome. Notably, literature data demonstrated that TBS and AFP have a synergistic impact on outcomes in these patients [22]. Moreover, in a recent study conducted by Tsilimigras [22], patients with similar values of TBS had different evolution, depending on AFP. We did not encounter any association between AFP and tumour burden calculated by SEC.

We consider that creating HCC prognostic schemas that include tumoral markers (such as AFP) can increase the predictive accuracy while optimizing patient selection for different therapies, thus improving the clinical benefit of a chosen therapeutic measure. The French Liver Transplantation Study Group previously suggested using AFP, tumour dimension and number in order to obtain a better selection of potential liver transplant recipients and, since its use, these AFP criteria demonstrated superiority to the Milan criteria when estimating the risk for HCC recurrence after transplant [23].

Some researchers have suggested the simultaneous assessment of other tumoral markers, in order to increase the sensitivity in HCC diagnosis and monitoring. One of these markers is AFP-L3, which is secreted in the early stages of HCC development and can therefore be used as an early HCC diagnostic tool [20,24]. It was revealed that in patients with AFP under 200 ng/ml, an AFP-L3 value higher than 35% of the total AFP is associated with specificity for HCC diagnosis that comes close to 100% [20,24]. The development of new technologies, including molecular biology, represents a new promising area concerning the research of new biomarkers associated with HCC.

It is important to mention that our research has several limitations, mainly given by the relatively small study group observed in a single centre and its retrospective observational character. However, we believe that we successfully brought into attention the importance of AFP in patients with HCC previously treated with DAAs, since these patients continue to represent a controversial type of cohort.

## Conclusions

We observed that serum AFP levels strongly correlate with HCC dimensions in patients previously treated with DAAs-a result that is in agreement with current literature data. Despite having suboptimal sensitivity, AFP remains a very important diagnostic and prognostic tool for HCC patients and simultaneous assessment of newer tumoral markers increases its strength. AFP not only correlates with TBS, but these two variables have a synergistic impact on outcomes. Creating prognostic algorithms that include tumoral markers can increase the predictive accuracy and improve the clinical benefit of a chosen therapeutic measure. With the continuous advances in molecular medicine, a new chapter in this domain has been opened and the research of newer, stronger biomarkers associated with HCC is a must, especially for AFP-negative tumours. We encourage incessant efforts concerning the discovery of new HCC biomarkers, while further bringing evidence to validate the currently existent ones.

## References

[1] Arnold M, Abnet CC, Neale RE, Vignat J, Giovannucci EL, McGlynn KA, Bray F (2020). Global burden of 5 major types of gastrointestinal cancer. Gastroenterology.

[2] Villanueva A (2019). Hepatocellular carcinoma. N Engl J Med.

[3] Iliescu EL, Toma L, Diaconu C (2020). Type 2 diabetes mellitus and the risk of hepatocellular carcinoma in chronic hepatitis C patients treated with direct acting antivirals. Proceedings of 6th International Conference on Interdisciplinary Management of Diabetes Mellitus and its Complications INTERDIAB.

[4] Kanwal F, Kramer J, Asch SM, Chayanupatkul M, Cao Y, El-Serag HB (2017). Risk of hepatocellular cancer in HCV patients treated with direct-acting antiviral agents. Gastroenterology.

[5] Bertino G, Ardiri A, Malaguarnera M, Malaguarnera G, Bertino N, Calvagno GS (2012). Hepatocellualar carcinoma serum markers. Semin Oncol.

[6] Carr BI, Akkiz H, Üsküdar O (2018). HCC with low- and normal-serum alpha-fetoprotein levels. Clin Pract (Lond).

[7] Worland T, Harrison B, Delmenico L, Dowling D (2018). Hepatocellular carcinoma screening utilising serum alpha-fetoprotein measurement and abdominal ultrasound is more effective than ultrasound alone in patients with non-viral cirrhosis. J Gastrointest Cancer.

[8] Gupta S, Bent S, Kohlwes J (2003). Test characteristics of alpha-fetoprotein for detecting hepatocellular carcinoma in patients with hepatitis C: a systematic review and critical analysis. Ann Intern Med.

[9] Gambarin-Gelwan M, Wolf DC, Shapiro R, Schwartz ME, Min AD (2000). Sensitivity of commonly available screening tests in detecting hepatocellular carcinoma in cirrhotic patients undergoing liver transplantation. Am J Gastroenterol.

[10] Agopian VG, Harlander-Locke MP, Markovic D (2017). Evaluation of patients with hepatocellular carcinomas that do not produce α-fetoprotein. JAMA Surg.

[11] Wong RJ, Ahmed A, Gish RG (2015). Elevated alpha-fetoprotein: differential diagnosis - hepatocellular carcinoma and other disorders. Clin Liver Dis.

[12] Isac T, Isac S, Ioanitescu S (2021). Dynamics of serum α-fetoprotein in viral hepatitis C without hepatocellular carcinoma. Exp Ther Med.

[13] European Association for the Study of the Liver, Clinical Practice Guidelines Panel: Chair, EASL Governing Board representative: Panel member (2020). EASL recommendations on treatment of hepatitis C: final update of the series. J Hepatol.

[14] Johnson PJ, Berhane S, Kagebayashi C (2015). Assessment of liver function in patients with hepatocellular carcinoma: a new evidence-based approach—the ALBI grade. J Clin Oncol.

[15] Hung YW, Lee IC, Chi CT (2021). Redefining tumor burden in patients with intermediate-stage hepatocellular carcinoma: the seven-eleven criteria. Liver Cancer.

[16] Serti E, Chepa-Lotrea X, Kim YJ (2015). Successful interferon-free therapy of chronic hepatitis C virus infection normalizes natural killer cell function. Gastroenterology.

[17] Abdelaziz AO, Nabil MM, Abdelmaksoud AH (2019). Tumor behavior of hepatocellular carcinoma after hepatitis C treatment by direct-acting antivirals: comparative analysis with non-direct-acting antivirals-treated patients. Eur J Gastroenterol Hepatol.

[18] El Kassas M, Elbaz T, Salaheldin M, Abdelsalam L, Kaseb A, Esmat G (2019). Impact of treating chronic hepatitis C infection with direct-acting antivirals on the risk of hepatocellular carcinoma: the debate continues - A mini-review. J Adv Res.

[19] Lin YH, Lin KH, Yeh CT (2020). Thyroid hormone in hepatocellular carcinoma: cancer risk, growth regulation, and anticancer drug resistance. Front Med.

[20] Hanif H, Ali MJ, Susheela AT, Khan IW, Luna-Cuadros MA, Khan MM, Lau DT (2022). Update on the applications and limitations of alpha-fetoprotein for hepatocellular carcinoma. World J Gastroenterol.

[21] Saffroy R, Pham P, Reffas M, Takka M, Lemoine A, Debuire B (2007). New perspectives and strategy research biomarkers for hepatocellular carcinoma. Clin Chem Lab Med.

[22] Tsilimigras DI, Hyer JM, Diaz A (2021). Synergistic impact of alpha-fetoprotein and tumor burden on long-term outcomes following curative-intent resection of hepatocellular carcinoma. Cancers.

[23] Duvoux C, Roudot-Thoraval F, Decaens T (2012). Liver transplantation for hepatocellular carcinoma: a model including α-fetoprotein improves the performance of Milan criteria. Gastroenterology.

[24] AlSalloom AA (2016). An update of biochemical markers of hepatocellular carcinoma. Int J Health Sci (Qassim).

